# Association of Hemoglobin Concentration and Its Change With Cardiovascular and All‐Cause Mortality

**DOI:** 10.1161/JAHA.117.007723

**Published:** 2018-01-29

**Authors:** Gyeongsil Lee, Seulggie Choi, Kyuwoong Kim, Jae‐Moon Yun, Joung Sik Son, Su‐Min Jeong, Sung Min Kim, Sang Min Park

**Affiliations:** ^1^ Department of Family Medicine Seoul National University Hospital Seoul Korea; ^2^ Department of Biomedical Sciences Seoul National University Hospital Seoul Korea

**Keywords:** anemia, hemoglobin, mortality, myocardial infarction, stroke, Myocardial Infarction, Cerebrovascular Disease/Stroke

## Abstract

**Background:**

Anemia is thought to increase mortality risks, but the effects of high hemoglobin concentration on survival are unclear. The effect of change in hemoglobin concentrations on survival in the general population is also unknown. This study aimed to examine the effect of hemoglobin concentrations and their changes on cardiovascular and all‐cause mortality risks.

**Methods and Results:**

We retrospectively analyzed a cohort from the NHIS‐HEALS (National Health Insurance Service–National Health Screening Cohort) database, including 170 078 men and 122 116 women without cardiovascular diseases, aged >40 years at baseline, with hemoglobin concentrations available for both first and second health examinations. We assessed 2 independent variables: “One‐time” hemoglobin concentrations and changes in hemoglobin from first to second examination. Participants were followed up for a median of 8 years to determine mortality related to myocardial infarction, stroke, all cardiovascular diseases, and all causes. Hemoglobin concentrations showed a U‐ or J‐shaped association with cardiovascular and all‐cause mortality after adjusting for cardiovascular risk factors. When anemic men achieved normal hemoglobin concentrations, the all‐cause mortality risk decreased, with an adjusted hazard ratio of 0.67 (95% confidence interval, 0.59–0.77), in comparison with those whose anemia persisted. Both increases and decreases of hemoglobin concentration outside the normal range elevated all‐cause mortality risk (adjusted hazard ratio: 1.39 [95% confidence interval, 1.28–1.49] and 1.10 [95% confidence interval, 1.01–1.20], respectively), compared with persistent normal hemoglobin concentrations. The trend was similar in women but was less significant.

**Conclusions:**

Low or high hemoglobin concentrations were associated with elevated cardiovascular and all‐cause mortality. Reaching and maintaining hemoglobin concentrations within the normal range correlated with decreased all‐cause mortality.

**Clinical Perspective**

**What Is New?**

- Low or high hemoglobin concentrations were associated with subsequently elevated 8‐year cardiovascular and all‐cause mortality in individuals in the general population aged >40 years, without cardiovascular disease at baseline.
- Reaching and maintaining hemoglobin concentrations within the normal range correlated with decreased subsequent 8‐year all‐cause mortality.

**What Are the Clinical Implications?**

- Our findings suggest that screening and management of hemoglobin concentrations in the middle‐aged and older adult population could contribute to lowering the risks of cardiovascular and all‐cause mortality.

## Introduction

A growing body of evidence suggests that anemia affects cardiovascular disease (CVD) and mortality in chronic kidney disease or heart failure patients and those undergoing maintenance hemodialysis.^1^ However, the effect of hemoglobin concentration on CVD in the general population is less clear. Chronic anemia could induce ventricular remodeling and cardiac dysfunction,^2^ thereby potentially increasing the risk of CVD or mortality. Because chronic anemia may be prevalent in the general population, particularly in women and in older adults,^3^ the effect of low hemoglobin concentrations on CVD‐related mortality in the general population needs to be further explored.

The effect of high hemoglobin concentrations on CVD‐related mortality is also unclear. Because red blood cells are the dominant determinants of blood viscosity, high hematocrit concentrations significantly slow blood flow throughout the body.^4^ Cigarette smokers are known to have higher concentrations of hemoglobin, which may increase the oxidative insults within the cell.^5^ Some studies have suggested that high hemoglobin concentrations or hematocrit could elevate the risk of CVD or mortality.^6^, ^7^ However, the effect of high hemoglobin concentrations on CVD varies across various subtypes of CVD.^8^ Furthermore, it is also currently unknown how the change of hemoglobin concentrations alters the risk of CVD‐related death. Whereas improving anemia could lower the risk of mortality in chronic kidney disease patients,^9^, ^10^ the effect of change in hemoglobin concentrations among those with low CVD risks is unclear.

In this study, we examined the association of hemoglobin concentration and its change with CVD and all‐cause mortality within the general population by studying a large Korean cohort from the NHIS‐HEALS (National Health Insurance Service–National Health Screening Cohort).

## Methods

The data, analytic methods, and study materials will not be made available to other researchers for purposes of reproducing the results or replicating the procedure.

### Study Overview

We analyzed data from the Korean NHIS‐HEALS database recorded from January 1, 2002, to December 31, 2013 (NHIS‐2017‐2‐457). The Korean national health examination is conducted biannually: Individuals who were born in an even year undergo screening in even years, and those who were born in odd years undergo a screening in odd years. The NHIS‐HEALS database covers all insurance claims data, and about 98% of Koreans are enrolled.^11^ Attrition over follow‐up in this database is known to be rare, given the nature of the national administration of the data, which is reported elsewhere.^12^ Data are collected from a population of ≈500 000 participants (10% of the entire population of those who underwent the national health examination provided by the NHIS) by simple random sampling and provided after deidentification.^12^ The NHIS‐HEALS database includes clinical data and the date of hospital visits, admissions, diagnoses, and death information. By law, all deaths must be reported to Statistics Korea. The NHIS database has been used for population‐based epidemiological studies, and its validity has been recognized elsewhere.^13^

### Study Population

From among the possible participants, 334 437 participants who were aged >40 years and who had undergone a medical examination with available hemoglobin concentrations for the first (2002 for those born in an even year, and 2003 for those born in an odd year) and second (2004 or 2005) health examinations were selected. We excluded 1036 participants who died and 40 989 participants who were diagnosed with myocardial infarction (MI) or stroke, based on diagnoses coded according to the *International Classification of Diseases, 10th revision (ICD‐10)* and questionnaires about their medical history before the index date (January 1, 2006). We also excluded 151 participants without sex values and 67 with hemoglobin <5 or ≥20 g/dL. Finally, 292 194 participants (170 078 men, 122 116 women) were included.

### Exposures and Covariates

**Figure 1:**
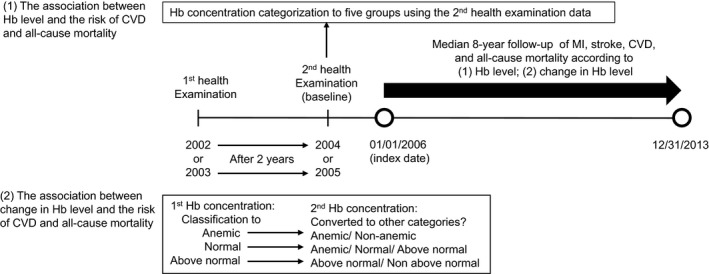
Timeline of the study. CVD indicates cardiovascular disease; Hb, hemoglobin; MI, myocardial infarction.

All participants underwent 2 hemoglobin (g/dL) measurements within a 2‐year period. Two sets of exposures were used (Figure 1). First, one‐time hemoglobin concentrations assessed before the index year were divided into 5 groups per sex and reflected the World Health Organization criteria for anemia (hemoglobin <13.0 and <12.0 g/dL in men and women, respectively).^14^ We did not use quintiles of hemoglobin concentration because the first quintile of men would be 5.0 to 13.9 g/dL, which could not distinguish anemic from normal status. Second, for establishing change, the hemoglobin concentrations at the first and second examinations were divided into 3 groups, with consideration of the criteria for anemia and highly abnormal concentrations of hemoglobin.

Covariates were based on the data from the second health examination and included age, sex, socioeconomic status, regular exercise (no, 1–2, 3–4, ≥5 times per week), smoking status (never and ever), alcohol use (none, <3, ≥3 times per week), body mass index (kg/m^2^), systolic and diastolic blood pressures (mm Hg), fasting serum glucose (mg/dL), total cholesterol (mg/dL), and Charlson Comorbidity Index (CCI). Although socioeconomic status and CCI are not typically considered major cardiovascular risk factors, they are regarded as risk factors for mortality. Effects of socioeconomic status on mortality or health status have been studied extensively.^15^ The CCI is the most commonly used comorbidity index for predicting mortality.^16^

### Main Outcomes

The main outcomes of the study were MI‐related, stroke‐related, CVD‐related, and all‐cause mortality that occurred from January 1, 2006, to December 31, 2013. *ICD‐10* codes were used to identify and classify the outcomes: MI (I21–I24), total stroke (I60–I69), and all CVD (I10–I99).

### Statistical Analyses

Continuous variables were expressed as mean (SD), and categorical variables were expressed as percentages and compared between groups using the Pearson χ^2^ test. Based on hemoglobin concentrations at the second health examination, the cohort was divided into 5 groups by sex: <13.0, 13.0 to 13.9, 14.0 to 14.9 (normal reference), 15.0 to 15.9, and ≥16.0 g/dL for men and <11.0, 11.0 to 11.9, 12.0 to 12.9 (normal reference), 13.0 to 13.9, and ≥14.0 g/dL for women. The criterion for the above‐normal range of hemoglobin concentration is generally 16.0 g/dL; however, the number of women with hemoglobin ≥16.0 g/dL was too low in the general population. For this reason, we used a hemoglobin concentration of 14.0 g/dL in women. Hazard ratios (HRs) and 95% confidence intervals for each outcome, based on the 5 groups of hemoglobin concentrations, were analyzed using Cox proportional hazards regression analyses. The multivariate‐adjusted analysis was adjusted for CVD risk factors, including age, socioeconomic status, body mass index, blood pressure, fasting serum glucose, total cholesterol, regular exercise, smoking status, alcohol use, and CCI. For subgroup analysis, the hemoglobin concentrations that had been divided into 5 groups were merged into 3 groups for each sex: <13.0, 13.0 to 15.9 (reference), and ≥16 g/dL for men and <12.0, 12.0 to 13.9 (reference), and ≥14.0 g/dL for women. Sensitivity analysis was performed by excluding patients diagnosed with cancer and chronic kidney disease.

Because change was rare (0.3%) in women in the group with a high hemoglobin concentration (≥16.0 g/dL at the first examination), we did not analyze the HR of change in high hemoglobin status among women. Statistical significance was set as a 2‐sided *P*<0.05. Data were collected using SAS 9.3 (SAS Institute), and statistical analyses were conducted using STATA 15.1 (StataCorp).

### Ethics

This study was conducted according to the guidelines in the Declaration of Helsinki, and all procedures involving human subjects (patients) were waived by the institutional review board of the Seoul National University (no. 1703‐039‐863). All participants were informed regarding the objective of the survey and provided consent. The NHIS database was anonymized according to strict confidentiality guidelines.

## Results

### Baseline Characteristics

The study population of 292 194 individuals (170 078 men and 122 116 women) was observed for a mean of 7.8 years (SD: 0.9), resulting in 2 279 113 person‐years of follow‐up. During follow‐up, there were 559 MI‐related, 936 stroke‐related, 1985 all‐CVD–related, and 12 677 all‐cause deaths.

Baseline characteristics for men and women are depicted in Table 1. The mean age was 54 years, and 42% of the participants were women. Current smokers (22% of total participants) were predominantly men. Participants without any comorbidities accounted for 35%. The mean hemoglobin concentrations of men and women were 14.8 g/dL (SD: 1.1) and 12.8 g/dL (SD: 1.1), respectively.

**Table 1:** General Characteristics of Participants According to Sex

	Total	Men	Women	*P* Value (Men vs Women)
Total, n	292 194	170 078	122 116	
Total, %	100	58.2	41.8	
Age (y), mean (SD)	54.2 (8.9)	53.6 (8.7)	55.1 (9.1)	<0.001
40–49, %	38.3	41.3	34.1	
50–64, %	35.2	34.9	35.5	
65–74, %	19.5	17.7	22.1	
≥75, %	7	6.2	8.2	
Socioeconomic status, %				<0.001
Upper	43.1	36.5	52.3	
Lower	56.9	63.5	47.7	
Smoking status, %				<0.001
Never smoker	64.8	43.7	94.2	
Ever smoker	31.2	51.87	2.4	
Alcohol use (per wk), %				<0.001
None	54.3	35	81.1	
<3 times	33.4	46.5	15.2	
≥3 times	10.7	17.3	1.5	
Regular exercise (per wk), %				<0.001
No exercise	48.8	42.6	57.4	
1–2 times	26.9	32.1	19.6	
3–4 times	12	13.4	10.1	
≥5 times	10.2	10.1	10.4	
CCI, %				<0.001
0	34.6	39.8	27.3	
1–2	49.9	46.9	54.1	
≥3	15.5	13.3	18.6	
BMI (kg/m^2^), mean (SD)	23.9 (2.9)	23.9 (2.8)	23.8 (3.0)	<0.001
SBP (mm Hg), mean (SD)	126.1 (17.0)	127.7 (16.4)	123.8 (17.5)	<0.001
DBP (mm Hg), mean (SD)	79.0 (11.1)	80.6 (10.9)	76.8 (11.1)	<0.001
FSG (mg/dL), mean (SD)	94.4 (28.3)	99.3 (30.3)	94.6 (25.0)	<0.001
Total cholesterol, mean (SD)	198.2 (36.6)	196.2 (36.0)	200.9 (37.3)	<0.001
Hemoglobin (g/dL), mean (SD)	14.0 (1.5)	14.8 (1.1)	12.8 (1.1)	<0.001
<11, %	2.3	0.4	4.9	
11 to <12, %	5.1	0.7	11.3	
12 to <13, %	17.2	3.4	36.4	
13 to <14, %	23	15.6	33.3	
14 to <15, %	24.8	34.3	11.7	
15 to <16, %	19	31.2	2.1	
≥16, %	8.5	14.4	0.3	

BMI indicates body mass index; CCI, Charlson comorbidity index; DBP, diastolic blood pressure; FSG, fasting serum glucose; SBP, systolic blood pressure.

### Association Between Baseline Hemoglobin Concentrations and Cardiovascular Mortality

**Figure 2:**
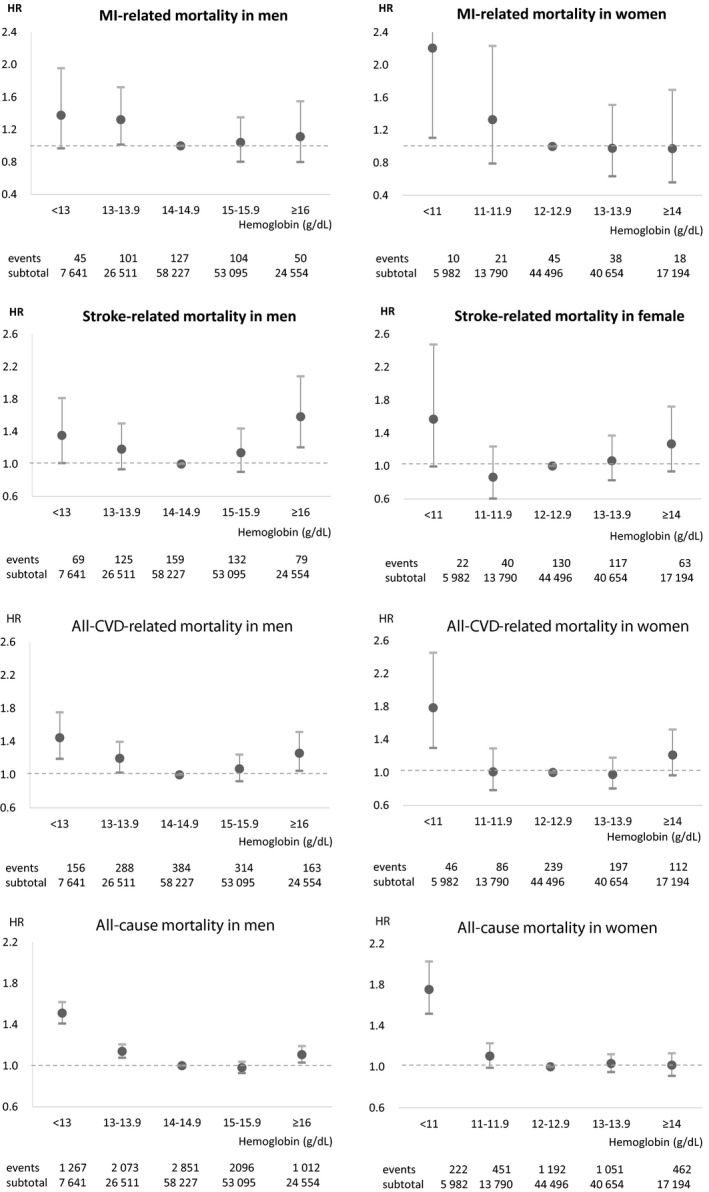
Association between hemoglobin concentration and cardiovascular and all‐cause mortality by sex. HR was calculated by Cox proportional hazards regression analysis adjusted for age, socioeconomic status, physical activity, smoking status, alcohol use, body mass index, blood pressure, fasting serum glucose, total cholesterol, and Charlson comorbidity index (95% confidence interval). *International Classification of Diseases, 10th Revision* codes were used to identify and classify the outcomes: MI (I21–I24), total stroke (I60–I69), and all CVD (I10–I99). CVD indicates cardiovascular disease; HR indicates hazard ratio; MI, myocardial infarction.

Figure 2 depicts the relationship between hemoglobin and mortality for each cardiovascular condition, according to sex, after adjusting for age, socioeconomic status, physical activity, smoking status, alcohol use, body mass index, blood pressure, fasting serum glucose, total cholesterol, and CCI. Lower hemoglobin concentrations were associated with increased MI‐related mortality, compared with those with hemoglobin concentrations between 14.0 and 14.9 g/dL for men and 12.0 and 12.9 g/dL for women. Both lower (men: <13.0 g/dL; women: <11.0 g/dL) and higher (men: ≥16.0 g/dL; women: ≥14.0 g/dL) hemoglobin concentrations were associated with stroke‐related, all‐CVD–related, and all‐cause mortality, with lower hemoglobin concentrations resulting in greater risks than higher hemoglobin concentrations. Additional analysis of the association of quintiles of hemoglobin concentrations with CVD‐related and all‐cause mortality among men and women is shown in Table S1, which was in line with categories based on clinical criteria.

These J‐ or U‐shaped associations between hemoglobin and mortality were also shown in subgroups divided by age, smoking status, and CCI (Table S2). The results of the sensitivity analysis, conducted by excluding patients with cancer (Table S3) or chronic kidney disease (Table S4), agreed with the main results.

### Association Between Change in Hemoglobin Concentrations and Cardiovascular and All‐Cause Mortality

**Figure 3:**
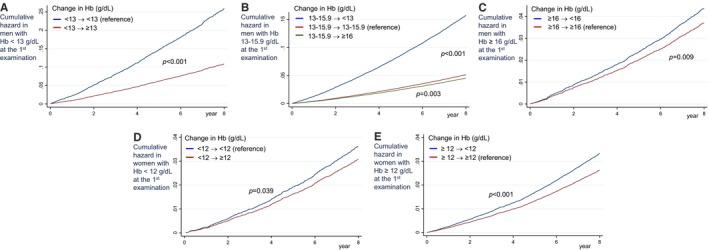
Unadjusted cumulative hazard curves for 8‐year all‐cause mortality by change in hemoglobin concentration status and by sex. Cumulative hazard based on first health examination: (A) men with Hb <13 g/dL, (B) men with Hb 13 to 15.9 g/dL, (C) men with Hb ≥16 g/dL, (D) women with Hb <12 g/dL, (E) women with Hb ≥12 g/dL. Hb indicates hemoglobin.

Figure 3 depicts unadjusted cumulative hazard curves for 8‐year all‐cause mortality by change in hemoglobin concentration status and by sex. All‐cause mortality with improving anemia was less than with persistent anemia. Conversely, all‐cause mortality with change in anemia was greater than with persistent normal hemoglobin. Tables 2 and 3 display the association between change in hemoglobin concentrations over a 2‐year period and each type of CVD mortality by sex after adjusting for CVD risk factors. Compared with men with persistent anemia (hemoglobin <13.0 g/dL), men with improved anemia status (hemoglobin ≥13.0 g/dL) had a decreased risk of all‐cause mortality (HR: 0.67 [95% confidence interval, 0.59–0.77]). Compared with men with persistent normal hemoglobin concentrations (hemoglobin: 13.0–15.9 g/dL), men with both decreasing and increasing hemoglobin concentrations had elevated risks of CVD mortality. In particular, reduced hemoglobin concentrations in men were associated with elevated CVD‐related and all‐cause mortality, and increased hemoglobin concentrations were associated with elevated all‐cause mortality. Compared with men with persistent hemoglobin concentrations above normal (hemoglobin ≥16.0 g/dL), men with decreased hemoglobin concentrations of ≥16.0 g/dL did not have increased risk of CVD mortality. Compared with women with persistent anemia (hemoglobin <12.0 g/dL), women with improved anemia (hemoglobin ≥12.0 g/dL) had decreased all‐cause mortality (HR: 0.80 [95% confidence interval, 0.68–0.93]). Conversely, compared with women with persistent normal hemoglobin, women with a change to anemic status had increased all‐cause mortality (HR: 1.13 [95% confidence interval, 1.01–1.26]).

**Table 2:** Association of Change in Hemoglobin Status With Cardiovascular Outcome and Mortality in Men

Men	Subtotal	MI Mortality		Stroke Mortality		CVD Mortality		All‐Cause Mortality	
		Events	aHR (95% CI)	Events	aHR (95% CI)	Events	aHR (95% CI)	Events	aHR (95% CI)
Hemoglobin <13 g/dL at the first examination									
At the second examination									
<13 g/dL	2064	16	1.00 (reference)	23	1.00 (reference)	58	1.00 (reference)	471	1.00 (reference)
≥13 g/dL	4538	18	0.71 (0.35–1.42)	34	1.00 (0.58–1.74)	68	0.75 (0.52–1.07)	468	0.67 (0.59–0.77)
Hemoglobin 13 to 15.9 g/dL at the first examination									
At the second examination									
<13 g/dL	5304	29	1.19 (0.80–1.76)	45	1.30 (0.94–1.78)	97	1.29 (1.04–1.60)	769	1.39 (1.28–1.49)
13–15.9 g/dL	117 099	287	1.00 (reference)	347	1.00 (reference)	833	1.00 (reference)	5882	1.00 (reference)
≥16 g/dL	13 938	33	1.11 (0.77–1.60)	49	1.29 (1.04–1.60)	101	1.21 (0.99–1.50)	618	1.10 (1.01–1.20)
Hemoglobin ≥16 g/dL at the first examination									
At the second examination									
≥16 g/dL	10 401	17	1.00 (reference)	27	1.00 (reference)	59	1.00 (reference)	377	1.00 (reference)
<16 g/dL	16 734	27	0.96 (0.52–1.77)	39	0.72 (0.44–1.19)	89	0.84 (0.60–1.17)	714	0.99 (0.85–1.10)

*International Classification of Diseases, 10th Revision* codes were used to identify and classify the outcomes: MI (I21–I24), total stroke (I60–I69), and all CVD (I10–I99). Hazard ratio was calculated by Cox proportional hazards regression analysis adjusted for age, socioeconomic status, physical activity, smoking status, alcohol use, body mass index, blood pressure, fasting serum glucose, and total cholesterol. aHR indicates adjusted hazard ratio; CI, confidence interval; CVD, cardiovascular disease; MI, myocardial infarction.

**Table 3:** Association of Change in Hemoglobin Status With Cardiovascular Outcome and Mortality in Women

Women	Subtotal	MI Mortality		Stroke Mortality		CVD Mortality		All‐Cause Mortality	
		Events	aHR (95% CI)	Events	aHR (95% CI)	Events	aHR (95% CI)	Events	aHR (95% CI)
Hemoglobin <12 g/dL at the first examination									
At the second examination									
<12 g/dL	8476	12	1.00 (reference)	28	1.00 (reference)	56	1.00 (reference)	302	1.00 (reference)
≥12 g/dL	10 627	10	0.63 (0.27–1.48)	31	0.87 (0.52–1.47)	61	0.83 (0.58–1.21)	321	0.80 (0.68–0.93)
Hemoglobin ≥12 g/dL at the first examination									
At the second examination									
≥12 g/dL	91 717	91	1.00 (reference)	279	1.00 (reference)	487	1.00 (reference)	2384	1.00 (reference)
<12 g/dL	11 296	19	1.56 (0.94–2.57)	34	0.86 (0.60–1.24)	76	0.80 (0.68–0.93)	371	1.13 (1.01–1.26)

*International Classification of Diseases, 10th Revision* codes were used to identify and classify the outcomes: MI (I21–I24), total stroke (I60–I69), and all CVD (I10–I99). Hazard ratio was calculated by Cox proportional hazards regression analysis adjusted for age, socioeconomic status, physical activity, smoking status, alcohol use, body mass index, blood pressure, fasting serum glucose, and total cholesterol. aHR indicates adjusted hazard ratio; CI, confidence interval; CVD, cardiovascular disease; MI, myocardial infarction.

## Discussion

In this representative, large, population‐based, retrospective, cohort study, hemoglobin concentrations showed a U‐ or J‐shaped association with CVD‐related and all‐cause mortality after adjusting for CVD risk factors. Similar results were obtained after excluding patients with chronic kidney disease or cancer.

### Baseline Hemoglobin Concentrations and CVD‐Related and All‐Cause Mortality

We found that stroke‐related, all‐CVD–related, and all‐cause mortality risks increased in both lower and higher hemoglobin concentrations, with lower hemoglobin concentrations showing a stronger increase in risk. In another cohort study of 21 829 participants with stable coronary artery disease, low hemoglobin concentrations were an independent predictor of mortality after 4 years.^17^ The participants consisted of patients with not only coronary artery disease but also MI, stroke, and heart failure. The authors did not find any significance at the highest quintile (hemoglobin >15.2 g/dL) in the total population, which contains the normal range of hemoglobin concentrations for men. Another study showed that anemia is an independent risk factor for CVD among 14 410 individuals from the general population after a follow‐up of 6.1 years.^18^ A retrospective cohort study with 11.2 years of follow‐up of 5888 community‐dwelling men and women showed that lower hemoglobin concentrations were associated with increased all‐cause mortality risk but not with CVD‐related mortality risk, which may be related to the relatively small number of CVD events.^19^

### Change in Hemoglobin Concentrations and CVD‐Related and All‐Cause Mortality

We found that achieving hemoglobin concentrations within the normal range could decrease the risk of all‐cause mortality, whereas deviating from the normal range could elevate the risk of all‐cause mortality. Few studies have evaluated the effects of change in hemoglobin over time in the general population. A study consisting of patients with coronary artery disease showed that persistent or new‐onset anemia is a predictor of cardiovascular mortality.^17^ A retrospective analysis of patients surviving to at least 6 months after MI events showed that the latest hemoglobin measurement had the highest prognostic power and that hemoglobin reduction was associated with an increased risk of all‐cause mortality.^20^ In our study, we also found that an increase in hemoglobin concentrations beyond the normal range was associated with increased all‐cause mortality, whereas achieving a normal hemoglobin level decreased the risk of all‐cause mortality.

### Possible Mechanisms

Several potential mechanisms could explain how low hemoglobin concentrations increase the risk of CVD‐related and all‐cause mortality. First, anemic status may result in ventricular remodeling and cardiac dysfunction. Chronic anemia with hemoglobin <10 g/dL is known to result in increased cardiac output that may lead to left ventricular hypertrophy,^2^ which is well‐noted among chronic kidney disease patients who are anemic.^21^ In our study, after excluding patients with chronic kidney disease, we found that anemia was associated with increased risk of CVD and mortality. Second, anemia may be a marker for an underlying inflammatory process, which would lead to increased risk of CVD events.^22^

The viscosity of blood is primarily determined by red blood cells. Greater hematocrit concentrations would thus significantly thicken the blood, slowing its flow rate throughout the body, raising the peripheral resistance, and reducing blood flow and perfusion to various tissues including the brain.^4^, ^23^ In addition, elevated hematocrit concentrations increase peripheral platelet activation and oxidative stress by releasing ADP in response to the accumulation of iron.^24^, ^25^ Increased hemoglobin concentrations in cigarette smokers, for example, has been suggested to perpetuate the oxidative insult within the cell further.^5^ When a subgroup analysis was performed by smoking status, we found that smokers with high hemoglobin (>16.0 g/dL) had a significantly increased risk of MI and all‐cause mortality compared with smokers with normal hemoglobin concentrations (13.0–15.9 g/dL). However, nonsmokers with high hemoglobin were also at significantly increased risk of CVD and mortality compared with nonsmokers with normal hemoglobin concentrations. Although we could not differentiate between the effect of cigarette smoking and high hemoglobin concentrations, the latter might be a risk factor for CVD and mortality regardless of smoking status.

### Study Limitations

This study has some limitations. First, abnormal hemoglobin concentrations in older adults may be caused by subclinical CVD rather than anemia with predisposition to CVD; however, we selected participants without any CVD at baseline to minimize this possibility. Second, chronic obstructive pulmonary disease could be a covariate that may be regarded as a possible comorbidity of anemia, simultaneously associated with smoking, which could have induced high hemoglobin concentration^26^, ^27^; however, we could not access separate comorbidity. Because of the retrospective study design, we did not fully capture these covariates; alternatively, we used the CCI, which is the most commonly used comorbidity index.^16^ Third, specific causes of anemia in these participants could be diverse and may have included iron‐deficiency anemia, anemia due to chronic disease, hemoglobinopathy, or prior gastrointestinal bleeding; however, we were unable to distinguish them. Last, although anemia could be related to inflammatory processes, according to a previous study,^22^ we could not assess inflammatory markers in this study. Further studies on whether hemoglobin concentration or its change is an independent risk factor for CVD, in which various covariates are considered, are required. Despite these limitations, the NHIS database is a nationwide representative database.^13^, ^24^ Furthermore, the 2 279 113 person‐years of follow‐up make this study the largest to date on hemoglobin concentrations, the change in hemoglobin concentrations, and CVD.

## Conclusions

We showed a J‐ or U‐shaped association between hemoglobin concentrations and cardiovascular mortality in both men and women after adjusting for CVD risk factors. Furthermore, we showed that achieving and maintaining hemoglobin concentrations within the normal range was related to decreased mortality in the general population.

## Author Contributions

Lee and Park conceptualized the study. Lee conducted the statistical analysis and wrote the first draft of the article. Choi, K. Kim, and Yun collected and organized data, and provided statistical analyses consultation. S. Kim, Son, and Jeong discussed the results. All authors approved submission of the final version of the article. K. Kim and S. Kim received a scholarship from the BK21‐plus education program provided by the National Research Foundation of Korea. We would like to thank the National Health Insurance Service for providing the database for research purpose (NHIS‐2017‐2‐457). We would like to thank Professor Seung‐Sik Hwang for his statistical expertise.

## Sources of Funding

This research was supported by Basic Science Research Program through the National Research Foundation (NRF) funded by the Ministry of Education (Grant No: 2017R1D1A1B03033721) in the Republic of Korea.

## Disclosures

None.

## Supporting information


**Table S1.** Association of Baseline Hemoglobin Status With Cardiovascular and All‐Cause Mortality by Sex
**Table S2.** Subgroup Analysis of Association Between Hemoglobin Concentration and Cardiovascular Mortality Stratified by Age, Smoking Status, and Charlson Comorbidity Index
**Table S3.** Sensitivity Analysis of Excluding Patients With Cancer
**Table S4.** Sensitivity Analysis of Excluding Patients With Chronic Kidney Disease
