# Supplementary material for: Association of Hemoglobin Concentration and Its Change With Cardiovascular and All‐Cause Mortality
Source: J Am Heart Assoc. 2018 Jan 29;7(3):e007723. doi: 10.1161/JAHA.117.007723 (PMC5850255; doi:10.1161/JAHA.117.007723)
Supplement: Supplementary file 1 — Table S1. Association of Baseline Hemoglobin Status With Cardiovascular and All‐Cause Mortality by Sex Table S2. Subgroup Analysis of Association Between Hemoglobin Concentration and Cardiovascular Mortality Stratified by Age, Smoking Status, and Charlson Comorbidity Index Table S3. Sensitivity Analysis of Excluding Patients With Cancer Table S4. Sensitivity Analysis of Excluding Patients With Chronic Kidney Disease [file JAH3-7-e007723-s001.pdf]

SUPPLEMENTAL MATERIAL

Table S1. Association of baseline hemoglobin status with cardiovascular and all-cause mortality according to sex.

Hemoglobin Quintile (g/dL)	Men					Women				
	Q1	Q2	Q3	Q4	Q5	Q1	Q2	Q3	Q4	Q5
	5.0-13.9	14.0-14.5	14.6-15.0	15.1-15.7	15.8-19.9	5.0-12.0	12.1-12.6	12.7-13.1	13.2-13.7	13.8-19.4
Total N	34,152	34,127	33,918	36,518	31,363	25,453	24,992	24,908	24,542	22,221
MI death	146	78	68	77	58	39	28	21	22	22
aHR	1.35	1.01	1.00	1.14	1.04	1.65	1.26	1.00	1.07	1.08
95% CI	1.01-1.81	0.73-1.39	reference	0.82-1.58	0.73-1.47	0.97-2.82	0.72-2.23	reference	0.59-1.94	0.59-1.96
Stroke death	194	108	85	81	96	81	65	79	67	80
aHR	1.20	1.04	1.00	0.99	1.48	0.91	0.79	1.00	0.88	1.11
95% CI	0.92-1.56	0.78-1.39	reference	0.73-1.35	1.10-1.98	0.66-1.24	0.57-1.09	reference	0.64-1.23	0.81-1.52
CVD death	444	246	208	208	199	174	119	134	115	138
aHR	1.24	1.01	1.00	1.02	1.20	1.13	0.84	1.00	0.89	1.12
95% CI	1.05-1.47	0.84-1.21	reference	0.84-1.23	0.99-1.46	0.90-1.42	0.66-1.08	reference	0.69-1.14	0.88-1.42
All death	3,340	1,790	1,499	1,419	1,251	857	655	635	644	587
aHR	1.26	1.02	1.00	0.98	1.09	1.22	0.98	1.00	1.04	1.01
95% CI	1.18-1.34	0.95-1.09	reference	0.92-1.06	1.02-1.18	1.10-1.35	0.88-1.09	reference	0.93-1.16	0.91-1.14

Abbreviations: Q, quintile; N, number; MI, Myocardial infarction; aHR, adjusted hazard ratio; CI, confidence interval, CVD, cardiovascular disease.

Hazard ratio was calculated by Cox proportional hazards regression analysis adjusted for age, socioeconomic status, physical activity, smoking status, alcohol habit, body mass index, blood pressure, fasting serum glucose, total cholesterol.

Table S2. Subgroup analysis of association between hemoglobin concentration and cardiovascular mortality stratified by age, smoking status, or CCI.

	Men			Women		
Hb concentration (g/dL)	<13	13-15.9	≥16	11-11.9	12-13.9	≥14
MI-related mortality						
Age: 40-64 years (N)	25630	98730	22596	16193	70238	14116
Events (n)	50	145	28	3	19	5
Adjusted HR	1.17	1.00	0.84	0.93	1.00	1.14
95% CI	0.84-1.63	reference	0.56-1.26	0.27-3.17	reference	0.42-3.07
Age: ≥65 years (N)	8522	12642	1958	3579	14912	3078
Events (n)	96	86	22	28	64	13
Adjusted HR	1.48	1.00	1.60	1.67	1.00	0.94
95% CI	1.09-2.00	reference	1.00-2.57	1.07-2.63	reference	0.52-1.72
Age: 40-49 years (N)	9951	48248	12016	8584	27955	5148
Events (n)	6	39	12	0	3	0
Adjusted HR	0.82	1.00	1.10	N/A	1.00	N/A
95% CI	0.35-1.95	reference	0.57-2.11		reference	
Age: ≥50 years (N)	24201	63124	12538	11188	57195	12046
Events (n)	140	192	38	31	80	18
Adjusted HR	1.36	1.00	1.06	1.58	1.00	1.04
95% CI	1.08-1.70	reference	0.75-1.51	1.04-2.40	reference	0.62-1.75
Smoking: never (N)	16390	48276	9612	18625	80269	16102
Events (n)	67	77	20	28	76	16
Adjusted HR	1.54	1.00	1.56	1.50	1.00	0.99
95% CI	1.10-2.17	reference	0.95-2.55	0.96-2.32	reference	0.58-1.71
Smoking: ever (N)	16088	58201	13926	431	1973	529
Events (n)	70	145	28	3	5	1
Adjusted HR	1.16	1.00	0.87	N/A	1.00	N/A
95% CI	0.86-1.56	reference	0.58-1.30		reference	
CCI=0 (N)	11951	45557	10219	5844	23068	4461
Events (n)	29	87	18	4	13	5
Adjusted HR	0.94	1.00	0.98	N/A	1.00	2.17
95% CI	0.61-1.45	reference	0.58-1.63		reference	0.75-6.33
CCI≥1 (N)	22201	65815	14335	13928	62082	12733
Events (n)	117	144	32	27	70	13
Adjusted HR	1.50	1.00	1.14	1.60	1.00	0.82
95% CI	1.16-1.93	reference	0.77-1.67	1.02-2.50	reference	0.45-1.49
Stroke-related mortality						

Age: 40-64 years (N)	25630	98730	22596	16193	70238	14116
Events (n)	47	127	38	14	63	23
Adjusted HR	1.17	1.00	1.35	1.20	1.00	1.64
95% CI	0.83-1.65	reference	0.94-1.95	0.67-2.16	reference	1.01-2.66
Age: ≥65 years (N)	8522	12642	1958	3579	14912	3078
Events (n)	147	164	41	48	184	40
Adjusted HR	1.17	1.00	1.61	0.91	1.00	1.08
95% CI	0.93-1.48	reference	1.14-2.27	0.66-1.26	reference	0.76-1.52
Age: 40-49 years (N)	9951	48248	12016	8584	27955	5148
Events (n)	8	25	5	7	13	2
Adjusted HR	1.60	1.00	0.74	1.83	1.00	N/A
95% CI	0.72-3.59	reference	0.28-1.94	0.73-4.61	reference	
Age: ≥50 years (N)	24201	63124	12538	11188	57195	12046
Events (n)	186	266	74	55	234	61
Adjusted HR	1.16	1.00	1.59	0.92	1.00	1.26
95% CI	0.95-1.41	reference	1.23-2.06	0.68-1.24	reference	0.95-1.68
Smoking: never (N)	16390	48276	9612	18625	80269	16102
Events (n)	93	119	37	53	227	56
Adjusted HR	1.15	1.00	2.07	0.93	1.00	1.22
95% CI	0.87-1.53	reference	1.43-3.00	0.69-1.26	reference	0.91-1.64
Smoking: ever (N)	16088	58201	13926	431	1973	529
Events (n)	92	158	40	4	8	6
Adjusted HR	1.22	1.00	1.18	N/A	1.00	2.98
95% CI	0.93-1.59	reference	0.83-1.67		reference	0.98-9.05
CCI=0	11951	45557	10219	5844	23068	4461
Events (n)	44	97	23	7	35	14
Adjusted HR	0.97	1.00	1.31	0.92	1.00	1.94
95% CI	0.67-1.41	reference	0.82-2.07	0.41-2.08	reference	1.04-3.64
CCI≥1	22201	65815	14335	13928	62082	12733
Events (n)	150	194	56	55	212	49
Adjusted HR	1.26	1.00	1.58	1.00	1.00	1.12
95% CI	1.01-1.57	reference	1.17-2.13	0.74-1.35	reference	0.82-1.53
CVD-related mortality						
Age: 40-64 years (N)	25630	98730	22596	16193	70238	14116
Events (n)	130	380	89	21	103	32
Adjusted HR	1.15	1.00	1.02	1.17	1.00	1.35
95% CI	0.94-1.41	reference	0.81-.129	0.73-1.87	reference	0.91-2.02

Age: ≥65 years (N)	8522	12642	1958	3579	14912	3078
Events (n)	314	318	74	111	333	80
Adjusted HR	1.29	1.00	1.49	1.18	1.00	1.19
95% CI	1.10-1.51	reference	1.16-1.92	0.95-1.47	reference	0.93-1.52
Age: 40-49 years (N)	9951	48248	12016	8584	27955	5148
Events (n)	20	93	26	8	16	2
Adjusted HR	1.10	1.00	1.02	1.80	1.00	N/A
95% CI	0.68-1.79	reference	0.66-1.58	0.76-4.22	reference	
Age: ≥50 years (N)	24201	63124	12538	11188	57195	12046
Events (n)	424	605	137	124	420	110
Adjusted HR	1.24	1.00	1.25	1.16	1.00	1.26
95% CI	1.09-1.41	reference	1.04-1.50	0.94-1.42	reference	1.02-1.56
Smoking: never (N)	16390	48276	9612	18625	80269	16102
Events (n)	211	267	66	117	398	96
Adjusted HR	1.30	1.00	1.55	1.16	1.00	1.18
95% CI	1.08-1.57	reference	1.18-2.03	0.94-1.43	reference	0.94-1.48
Smoking: ever (N)	16088	58201	13926	431	1973	529
Events (n)	208	399	93	8	21	12
Adjusted HR	1.19	1.00	1.06	1.87	1.00	1.87
95% CI	1.00-1.41	reference	0.84-1.33	0.80-4.37	reference	0.90-3.88
CCI=0	11951	45557	10219	5844	23068	4461
Events (n)	95	242	47	15	64	23
Adjusted HR	0.97	1.00	0.98	1.12	1.00	1.68
95% CI	0.76-1.24	reference	0.72-1.35	0.64-1.98	reference	1.04-2.73
CCI≥1	22201	65815	14335	13928	62082	12733
Events (n)	349	456	116	117	372	89
Adjusted HR	1.35	1.00	1.34	1.20	1.00	1.15
95% CI	1.17-1.57	reference	1.09-1.65	0.97-1.49	reference	0.91-1.45
All-cause mortality						
Age: 40-64 years (N)	25630	98730	22596	16193	70238	14116
Events (n)	1254	2756	623	203	869	181
Adjusted HR	1.37	1.00	1.06	1.16	1.00	0.97
95% CI	1.28-1.46	reference	0.97-1.16	0.99-1.35	reference	0.83-1.14
Age: ≥65 years (N)	8522	12642	1958	3579	14912	3078
Events (n)	2086	2191	389	470	1374	281
Adjusted HR	1.18	1.00	1.20	1.23	1.00	1.02
95% CI	1.11-1.26	reference	1.07-1.33	1.11-1.37	reference	0.90-1.16

Age: 40-49 years (N)	9951	48248	12016	8584	27955	5148
Events (n)	218	696	188	66	202	29
Adjusted HR	1.35	1.00	1.10	1.10	1.00	0.73
95% CI	1.16-1.58	reference	0.94-1.30	0.84-1.46	reference	0.49-1.08
Age: ≥50 years (N)	24201	63124	12538	11188	57195	12046
Events (n)	3,122	4,251	824	607	2,041	433
Adjusted HR	1.25	1.00	1.12	1.23	1.00	1.03
95% CI	1.19-1.31	reference	1.03-1.20	1.12-1.35	reference	0.93-1.14
Smoking: never (N)	16390	48276	9612	18625	80269	16102
Events (n)	1522	2020	370	606	2008	411
Adjusted HR	1.27	1.00	1.14	1.25	1.00	1.01
95% CI	1.18-1.36	reference	1.02-1.28	1.14-1.37	reference	0.91-1.13
Smoking: ever (N)	16088	58201	13926	431	1973	529
Events (n)	1621	2674	598	37	150	41
Adjusted HR	1.26	1.00	1.10	1.07	1.00	1.00
95% CI	1.18-1.34	reference	1.01-1.21	0.74-1.54	reference	0.70-1.42
CCI=0	11951	45557	10219	5844	23068	4461
Events (n)	652	1432	298	86	383	82
Adjusted HR	1.12	1.00	1.09	1.01	1.00	1.09
95% CI	1.02-1.23	reference	0.96-1.24	0.80-1.28	reference	0.86-1.39
CCI≥1	22201	65815	14335	13928	62082	12733
Events (n)	2688	3515	714	587	1860	380
Adjusted HR	1.30	1.00	1.12	1.28	1.00	0.98
95% CI	1.24-1.37	reference	1.03-1.21	1.17-1.41	reference	0.88-1.10

Abbreviation: HR, hazard ratio.

Hazard ratio calculated by Cox proportional hazards regression analysis adjusted for age, socioeconomic status, physical activity, smoking status, alcohol habit, body mass index, blood pressure, fasting serum glucose, total cholesterol.

Table S3. Sensitivity analysis of excluding patients with cancer.

	Men					Women				
Hb concentration (g/dL)	<13	13-13.9	14-14.9	15-15.9	≥16	<11	11-11.9	12-12.9	13-13.9	≥14
Excepting patients with cancer, N	6815	25035	55841	51170	23744	5683	13125	42456	38877	16418
MI-related mortality, n	42	88	122	98	47	10	21	41	35	16
Adjusted HR	1.46	1.23	1.00	1.00	1.06	2.48	1.46	1.00	1.00	0.97
95% CI	1.02-2.10	0.94-1.63	reference	0.77-1.31	0.75-1.48	1.23-4.97	0.86-2.47	reference	0.63-1.57	0.54-1.74
Stroke-related mortality, n	62	116	141	124	76	21	37	123	108	62
Adjusted HR	1.45	1.26	1.00	1.19	1.68	1.63	0.84	1.00	1.04	1.33
95% CI	1.06-1.97	0.98-1.62	reference	0.94-1.52	1.27-2.23	1.02-2.60	0.58-1.22	reference	0.81-1.36	0.98-1.82
All-CVD-related mortality, n	141	262	356	299	157	43	79	222	183	106
Adjusted HR	1.51	1.20	1.00	1.09	1.28	1.86	0.99	1.00	0.98	1.24
95% CI	1.23-1.84	1.02-1.41	reference	0.93-1.27	1.06-1.55	1.34-2.58	0.77-1.29	reference	0.81-1.20	0.98-1.57
All-cause mortality, n	985	1756	2484	1891	924	183	397	1047	940	411
Adjusted HR	1.46	1.13	1.00	1.00	1.13	1.73	1.10	1.00	1.05	1.04
95% CI	1.35-1.58	1.06-1.20	reference	0.94-1.06	1.05-1.22	1.48-2.03	0.98-1.24	reference	0.96-1.15	0.92-1.16

Abbreviations: Hb, hemoglobin; CKD, chronic kidney disease; MI, myocardial infarction; CVD, cardiovascular disease.

Table S4. Sensitivity analysis of excluding patients with chronic kidney disease.

	Men					Women				
Hb concentration (g/dL)	<13	13-13.9	14-14.9	15-15.9	≥16	<11	11-11.9	12-12.9	13-13.9	≥14
Excepting patients with CKD, N	7526	26445	58200	53032	24524	5943	13751	44432	40605	17175
MI-related mortality, n	45	101	127	104	49	10	21	45	38	18
Adjusted HR	1.40	1.32	1.00	1.04	1.09	2.21	1.34	1.00	0.98	0.97
95% CI	0.98-1.98	1.02-1.73	reference	0.80-1.35	0.78-1.52	1.11-4.42	0.79-2.25	reference	0.63-1.51	0.56-1.69
Stroke-related mortality, n	66	125	159	132	79	22	40	130	117	63
Adjusted HR	1.31	1.18	1.00	1.14	1.59	1.58	0.87	1.00	1.07	1.27
95% CI	0.97-1.76	0.93-1.50	reference	0.90-1.44	1.21-2.08	1.00-2.49	0.61-1.24	reference	0.83-1.37	0.93-1.72
All-CVD-related mortality, n	150	288	384	314	162	46	86	237	197	112
Adjusted HR	1.41	1.20	1.00	1.07	1.25	1.81	1.02	1.00	0.99	1.22
95% CI	1.16-1.71	1.03-1.40	reference	0.92-1.24	1.04-1.51	1.32-2.49	0.80-1.31	reference	0.81-1.19	0.97-1.53
All-cause mortality, n	1238	2063	2846	2093	1009	212	447	1190	1046	462
Adjusted HR	1.50	1.14	1.00	0.98	1.11	1.69	1.10	1.00	1.03	1.02
95% CI	1.40-1.60	1.07-1.20	reference	0.93-1.04	1.03-1.19	1.45-1.95	0.99-1.23	reference	0.95-1.12	0.91-1.14

Abbreviations: Hb, hemoglobin; CKD, chronic kidney disease; MI, myocardial infarction; CVD, cardiovascular disease.
